# Associations between personality and musculoskeletal disorders in the general population: A systematic review protocol

**DOI:** 10.3389/fpsyt.2022.1079162

**Published:** 2023-01-25

**Authors:** Shae E. Quirk, Heli Koivumaa-Honkanen, Risto J. Honkanen, Mohammadreza Mohebbi, Bianca E. Kavanagh, Jeremi Heikkinen, Lana J. Williams

**Affiliations:** ^1^School of Medicine, Institute for Mental and Physical Health and Clinical Translation, Deakin University, Geelong, VIC, Australia; ^2^Institute of Clinical Medicine, Psychiatry, University of Eastern Finland, Kuopio, Finland; ^3^Kuopio Musculoskeletal Research Unit (KMRU), Institute of Clinical Medicine, University of Eastern Finland, Kuopio, Finland; ^4^Mental Health and Wellbeing Center, Kuopio University Hospital, Kuopio, Finland; ^5^Biostatistics Unit, Faculty of Health, Deakin University, Geelong, VIC, Australia; ^6^Barwon Health, University Hospital Geelong, Geelong, VIC, Australia

**Keywords:** systematic review, personality disorder, comorbidity, musculoskeletal diseases, musculoskeletal disorders, MSD

## Abstract

There is growing evidence of the comorbidity between personality disorder (PD) and musculoskeletal disorders (MSDs). However, there are no systematic reviews including critical appraisal and meta-analyses that identify, evaluate, and synthesize the available evidence on these associations. Therefore, we present here a protocol of the methodology to undertake a systematic review, with the objective to evaluate associations between PD and MSDs in epidemiological population-based studies. A systematic review of observational studies will be conducted. A complete search strategy will be developed in consultation with a health librarian. To identify peer-reviewed literature, the search will be translated for, and implemented in Medline Complete, CINAHL Complete, and PsycINFO *via* the EBSCOhost platform from 1990 to the present. Gray literature will be identified. Studies will be eligible if they examine general population participants aged 15 years and over. Associations of interest are the presence of threshold or positive screen according to the DSM-V/5 (groupings: any, Clusters A, B, C, specific PD) or ICD-10 for PD in relation to arthritis, back/neck conditions, fibromyalgia, osteopenia/osteoporosis, and/or “any” of these MSDs. Data extraction and critical appraisal will be conducted in line with the Joanna Briggs Institute (JBI) guidance for systematic reviews of etiology and risk. The results from all studies will be presented in tables, text, and figures. A descriptive synthesis will present the characteristics of included studies, critical appraisal results, and descriptions of the main findings. Where appropriate, meta-analyses will be performed. If heterogeneity (e.g., *I*^2^ = 50%) is detected, subgroup/sensitivity analysis may be used to explore the possible sources. The systematic review does not require ethics approval. The proposed systematic review will strengthen the evidence base on what is known regarding associations between PD and MSDs by identifying, evaluating, and synthesizing the findings of existing observational studies including meta-analyses, where appropriate.

## 1. Introduction

Separately, mental disorders and musculoskeletal disorders (MSDs) are the two main causes of years lived with disability (YLD) (1), and still, their comorbidities are largely neglected in research and practice (2). There is increasing awareness of plausible associations between MSDs and personality disorder (PD). We previously undertook a scoping review, which examined a range of MSDs including conditions of the back, joints, soft tissue, and conditions of bone density and structure in relation to PD (3). Of note and interest, it revealed associations between PD and specific MSDs including arthritis, chronic neck/back pain, fibromyalgia, and reduced bone mineral density (4). We recommended further research, including the conduct of systematic reviews and meta-analyses to strengthen the evidence base in this field. Building on this prior work, we plan to undertake a systematic review on population-based associations between PD and MSDs, undertake critical appraisal of the identified evidence sources, and conduct meta-analyses, where appropriate. The ensuing review may lead to increased understanding of the levels of evidence on this topic, and improve awareness of these comorbidities in the community.

Traditionally, there were 10 distinct categorical PDs (organized into Clusters A, B, and C depending on typical features of the disorders). However, the field is also moving toward a unitary construct of PD for the International Classification of Diseases 11th Revision (ICD-11) (5). Often beginning earlier in life, PD is characterized by difficulties with interpersonal relating and adaptive functioning (6). The difficulties are apparent in patterns of thinking, emotional experiences, behaviors and coping mechanisms—appearing in a range of important areas including social situations (e.g., relationships/dynamics with family, friends, peers, or partners), and education and occupational settings. People presenting with PD pathology (i.e., below diagnostic thresholds or those who “screen positive”) also experience these difficulties, to varying extents, compared to people without (7). PD is common, with approximately one in eight people residing in Western countries estimated to have a PD (8), and is associated with a broad range of chronic physical illnesses (9, 10).

Elsewhere, the population prevention and management of MSDs (11), and separately, depression, anxiety, and other common mental disorders (12, 13) are increasingly recognized by intergovernmental initiatives as targets for intervention. There is growing awareness of the need for better integration and management of these comorbidities (2, 14, 15). However, PD has not yet gained a proportional public health awareness as a common mental health disorder, nor in relation to health. Consequently, others have highlighted that there are still limited evidence-based approaches and interventions aimed to improve the health of people with PD (16), which is especially the case concerning MSDs. In part, this may be due to a lack of systematic reviews incorporating evidence from population-based epidemiological studies, and using robust methodologies to evaluate the current evidence.

Existing descriptive and narrative reviews have made valuable contributions to the literature by summarizing associations between PD and diverse physical health conditions, along with proposing their mechanistic links and prompting further research in the field (9, 10, 17–20). While it is acknowledged that existing reviews may employ different approaches, given their varying aims, there are differences in the level/quality of reporting on searching and selecting articles, and extracting, analyzing, and presenting results of existing reviews, including a lack of meta-analyses. With a focus on MSDs specifically, the proposed systematic review will build on these previous efforts by employing a rigorous approach to selecting, performing critical appraisal, and synthesizing the available evidence including meta-analyses, where appropriate.

Therefore, we present a protocol of the methodology to undertake a systematic review, with the objective to evaluate population-based epidemiological associations between PD and the following MSDs: arthritis, back/neck pain, fibromyalgia/muscular pain, and osteopenia/osteoporosis.

The research questions guiding this review are as follows:

1. Is PD associated with an increased risk of arthritis, back/neck pain, fibromyalgia/muscular pain, and osteopenia/osteoporosis and/or “any” of these conditions compared people without PD?
2. For the question above, what methodological characteristics explain the heterogeneity in results?

## 2. Methods and analyses

### 2.1. Design

This protocol is registered with PROPSERO: CRD42021243094 and was developed in accordance with the Preferred Reporting Items for Systematic review and Meta-Analysis Protocols (PRISMA-P) (21) and the guidance published by the Joanna Briggs Institute (JBI) for conducting systematic reviews of etiology and risk (22).

### 2.2. Inclusion criteria

The Population, Exposure, Outcome (PEO) inclusion criteria (22) are presented as follows:

#### 2.2.1. Population

Studies will be considered if they examine general population participants aged 15 years and over. Other than age, there will be no specific exclusions based on any participant characteristics.

#### 2.2.2. Exposure

The exposure(s) of interest include the presence of categorical PD according to:

• DSM-IV/5 or ICD-10 criteria; and
• Assessed by a structured/semi-structured interview—administered by a trained interviewer (i.e., graduate with a relevant qualification or lay interviewer) or expert (i.e., relevant health professional)—or screening instruments.

As priority, we will classify PD according to the following separate groupings:

• “Any” categorical PD
• Clusters A, B, or C PDs
• Specific PDs
• PD “pathology,” PD “positive screen” or “probable” PD.

Subsequently, these groupings may be further combined into an overall “any” PD category, which we anticipate may be more feasible to analyze. Details regarding the measurement of PD (e.g., diagnosis, classification, and administration) will be extracted to inform potential subgroup analyses.

#### 2.2.3. Outcomes

The primary outcome(s) are the presence (yes/no) of one or more of the following MSDs:

• Arthritis.
• Back/neck pain.
• Fibromyalgia/muscular pain.
• Osteopenia/osteoporosis.
• Any of these conditions.

Studies will be eligible if they assess/identify one or more of the above outcomes(s) according to:

• ICD-10 criteria, diagnosed by a relevant health professional, or other relevant clinical criteria reported in linked medical records (i.e., “expert diagnosis”).
• Self-reported from questionnaire responses or semi-structured interviews (i.e., “self-report”).

If an individual study reports on more than one MSD, all relevant analyses will be included. We will extract the diagnosis and definitions of MSDs including the assessment method (expert diagnosis/self-report), which are anticipated to vary between studies. Each relevant condition will be considered regardless of “current,” “12-month,” or “lifetime” status.

These MSDs have been selected as outcomes of interest for this review, as recent scoping work has identified them as conditions that may be highly comorbid with PDs in clinical and/or general populations (i.e., arthritis, back/neck pain, fibromyalgia), or there is emerging evidence of their associations (i.e., poorer bone health) (3, 4).

#### 2.2.4. Study designs

Studies will be considered eligible if they are population-based, observational studies including cross-sectional (analytical), case-control, or cohort studies. There will be no restrictions on length of follow-up for longitudinal studies.

#### 2.2.5. Language

Google Translate may be utilized if potentially relevant sources are identified that are published in languages other than English. However, it is acknowledged that Google Translate may not be appropriate for some languages. Translators may be considered depending on the number of articles retrieved that are published in languages other than English and constraints (i.e., time and costs).

### 2.3. Exclusions

The following exclusion criteria will be applied:

• Studies with a non-eligible design (i.e., intervention study designs, qualitative study designs, descriptive study designs).
• Participants under the minimum age of 15 years.
• Does not examine PD according to the inclusion criteria.
• Does not examine MSDs according to the inclusion criteria (i.e., examined other diseases/conditions).
• Wrong context/setting (i.e., primary/secondary/tertiary/emergency care, prisons/correctional or other specialized/clinical settings).

### 2.4. Information sources

Database searching will be used to identify peer-reviewed journal articles that meet the inclusion criteria. The authors of the studies considered eligible may be contacted to make data clarifications/requests (e.g., depending on the nature of the query, and time and resource constraints). Information sources will be restricted to those published on or after the ICD-10 was endorsed by Forty-third World Health Assembly in 1990.

In addition, gray literature that meets the inclusion criteria—such as dissertations, or reports that describe findings from population health surveys initiated by governments/research agencies or other experts that undertake research on behalf of relevant agencies—will be considered. Additional information sources may be identified using “snowballing” techniques, including screening and reviewing reference lists of eligible studies. Complete details regarding information sources will be provided in the review.

### 2.5. Search strategy

First, to confirm no prior systematic review has been published that addresses our objectives, we conducted a preliminary search on 10 June 2021 in PROSPERO, PubMed, the Cochrane Database of Systematic Reviews, and JBI Evidence Synthesis.

An indicative search was developed and conducted in Medline Complete using the EBSCOhost platform on 26 August 2021, yielding 236 results (see Supplementary Table 1). A complete search strategy will be developed in consultation with a health librarian. It may be further refined using additional Index terms/keywords, and using Boolean operators, truncations, and explode functions (where appropriate). The Medline Complete search will be translated for Embase, and CINAHL Complete and PsycInfo databases. The final search strategy will be evaluated by a health librarian using the Peer Review of Electronic Search Strategies (PRESS) checklist. First, gray literature will be searched using an adapted search for the CORDIS and ProQuest databases, and second, in Google (if further gray literature searching is deemed warranted). The complete details regarding the development of the search strategy and results will be prepared as Supplementary material and submitted with the final review.

### 2.6. Data management

One reviewer will implement the search strategy and manage the records. The records from the combined searches will be exported to a reference management software such as Covidence with duplicates removed (23). Extracted data will be entered into a fit-for-purpose excel file, and analysis will be performed using the statistical analyses program, Stata.

### 2.7. Selection process

#### 2.7.1. Article selection tool

A selection aid will be developed to enhance the accurate identification and selection of the citations. It will be tested by at least two reviewers. Good agreement will be determined if the two reviewers achieve a consensus rate of 75% based on the screening decisions (include/exclude) and reasons for exclusion on a sample of 5% of the records. If there are discrepancies of 75% or greater, the reviewers will consider modifications to the inclusion criteria and report these deviations in the main review.

#### 2.7.2. Screening

Two reviewers will screen titles/abstracts and review full-text articles, independent from each other using Covidence. Any discrepancies at the screening or full-text stage will be resolved by the two reviewers in the first instances and/or a consensus discussion with the supervising authors. Reasons for exclusion will be provided for the full-text screening stage.

In terms of articles identified by “snowballing,” the reference lists of selected articles will be hand-searched using the backward approach by one reviewer. In the first instance, studies will be screened for relevance based on their titles. If further detail is required, the reviewer will access the abstract and/or full-text article.

The final list of articles/gray literature will be confirmed against the inclusion criteria by at least the second reviewer and/or the supervising author.

### 2.8. Data collection process

#### 2.8.1. Critical appraisal of individual studies

Two reviewers will critically appraise the selected studies using standardized critical appraisal checklists developed by JBI, independently. The JBI critical appraisals tools were selected as they offer a means to assess the methodological quality of observational studies (including bespoke tools for each cohort, cross-sectional, and case-control designs) such as the possible, or extent of bias deriving from the design, conduct, and/or analysis of studies. Any potential disagreements will be solved by consensus between the two reviewers and/or the supervising author. The methodological quality of individual studies will be reported in text/tables.

The Grading of Recommendations, Assessment, Development and Evaluation (GRADE) approach will be used to assess the certainty of the evidence, pending availability and appropriateness of the observational studies selected for the review (24).

#### 2.8.2. Data extraction

A data extraction tool will be developed and refined in consultation with a statistician on the review team (MM). The indicative data items are appended to this protocol as Supplementary material (see Supplementary Table 2). It is intended to capture key data items that are required to address the research objectives including generic citation details, study and participant characteristics, assessment of PD and MSDs, and main results. These data items were determined *a priori* including considerations given to known differences in methodological approaches for the assessment of PD, which may influence associations across different studies. Where feasible, two reviewers will undertake data extraction, independently. A consensus meeting will be held between the same reviewers and the supervising author to resolve and correct potential discrepancies.

#### 2.8.3. Outcomes and prioritization

The primary outcome(s) are the categorical (yes/no) presence of each specific MSD in relation to the PD groupings. The secondary outcome is the categorical (yes/no) presence of any “pooled” MSDs from the identified studies. For models with the highest number of confounding adjustments, ORs, RRs (risk ratio), and 95% confidence intervals (CIs) will be extracted.

### 2.9. Data synthesis and analysis

#### 2.9.1. Narrative synthesis

A narrative synthesis will present the characteristics of included studies, critical appraisal results, and descriptions of the main findings in text and tables/figures. Where possible, the narrative synthesis will be summarized according to each MSD of interest. The results will also be visually presented using EPPI-Mapper.

#### 2.9.2. Meta-analysis

Where appropriate, a quantitative synthesis will be performed with the odds ratio being considered the main effect size for binary outcomes. Risk Ratios (RR) from relevant studies will be transformed into ORs using a predetermined method (25). ORs/RRs with 95% CI for all categories of PD/MSDs will be extracted for the analysis. As potential heterogeneity is anticipated, all analyses will be conducted in Stata 17 using random-effects models. The OR estimate from the most fully adjusted models from each report will be used in the pooled analysis. Complete information regarding the analyses will be presented in the final review.

The results will be presented graphically in a forest plot (for each grouping where appropriate). Heterogeneity will be explored using the *I*^2^ statistic—where appropriate. If significant heterogeneity is detected, subgroup analysis by the exclusion of one study at a time will be performed to assess the stability of results and potential sources of heterogeneity. Subgroup analyses may also be performed to check for potential source of heterogeneity according to study design, study quality, sex, study location, and/or adjustment for important confounding factors.

If a quantitative synthesis is deemed inappropriate for all of, or for specific planned groupings, the authors will provide reasons and justifications for presenting the findings as a narrative synthesis and in tables/figures.

#### 2.9.3. Additional analyses

While the proposed comprehensive search strategy may minimize the potential for publication bias, publication bias will be formerly assessed by visually inspecting funnel plots.

### 2.10. Presenting and reporting results

PRISMA and the Meta-analysis of Observational Studies in Epidemiology (MOOSE) guidelines (26) will be adhered to for the conduct and reporting of the findings of the review. A PRISMA flow diagram will be used for reporting the screening and selection process including the numbers and reasons for exclusions (full-text stage only). The discussion will include a summary of the major findings, limitations of the included studies and review, and mechanisms/clinical implications.

## 3. Discussion

This protocol was developed to adhere to relevant guidance including the PRISMA-P guidelines. The proposed systematic review will strengthen the evidence base on what is known regarding associations between PD and MSDs by evaluating the findings of existing observational studies including conducting meta-analyses, where possible. In terms of possible limitations, there is the potential for inconsistent quality in the conduct and reporting of observational studies that will be included in the review.

## 4. Conclusion

This protocol presented the methodology to undertake a systematic review on associations between PD and MSDs among people in the general population.

## Ethics statement

The findings will be published in a peer-reviewed scientific journal and presented at conferences relevant to the field. The published findings from the review will be disseminated to existing networks. This study is exempt from ethics approval or consent procedures, as it does not involve the inclusion of identifiable human data.

## Author contributions

SEQ, HK-H, and LJW conceptualized and designed the protocol. MM provided statistical guidance. All authors provided input into the methodology, significantly contributed to drafting the article, and approved the final version to be published.
