# Supplementary material for: Associations between personality and musculoskeletal disorders in the general population: A systematic review protocol
Source: Front Psychiatry. 2023 Jan 25;13:1079162. doi: 10.3389/fpsyt.2022.1079162 (PMC9905843; doi:10.3389/fpsyt.2022.1079162)
Supplement: Supplementary file 1 [file Data_Sheet_1.docx]

**Appendix Table 1**: *Preliminary search in Medline Complete*

| Search # | Search terms | Results |
| --- | --- | --- |
| S9 | S7 AND S8 | 236 (Limiters - Date of Publication: 19900101-20211231)  332 |
| S8 | S3 OR S4 OR S5 OR S6 | 1,211,457 |
| S7 | S1 OR S2 | 53,550 |
| S6 | MH ("Fibromyalgia" OR “Myalgia” OR “Musculoskeletal Pain”) OR TI (fibromyal* OR "musc* pain" OR “muscle aches” OR “muscle soreness”) OR AB (fibromyal* OR "musc* pain" OR “muscle aches” OR “muscle soreness”) | 27,964 |
| S5 | MH ("Osteoporosis+") OR TI (osteoporosis OR osteopenia OR bone) OR AB (osteoporosis OR osteopenia OR bone) | 789,184 |
| S4 | MH ("Back Pain+" OR “Neck Pain") OR TI (“back pain” OR backache* OR “low back pain” OR “neck pain” OR "lumbar pain" OR "spinal pain" OR “spine pain” OR "spinal stenosis" OR "lumbar stenosis" OR "intervertebral disc displacement" OR “disc herniation” OR sciatica) OR AB (“back pain” OR backache* OR “low back pain” OR “neck pain” OR "lumbar pain" OR "spinal pain" OR “spine pain” OR "spinal stenosis" OR "lumbar stenosis" OR "intervertebral disc displacement" OR “disc herniation” OR sciatica) | 92,325 |
| S3 | MH (Arthritis+) OR TI (arthritis OR osteoarthritis OR "osteo-arthritis" OR "osteoarthritic") OR AB (arthritis OR osteoarthritis OR "osteo-arthritis" OR "osteoarthritic") | 353,719 |
| S2 | MH ("Personality Disorders+") | 42,564 |
| S1 | TI ((personality OR borderline) N2 (disorder* OR dysfunction* OR pathology OR feature* OR symptom*)) OR AB ((personality OR borderline) N2 (disorder* OR dysfunction* OR pathology OR feature* OR symptom*)) | 25,801 |

Note: Search modes = Boolean/Phrase. Search fields = search in abstract field (AB); search in MeSH/Index Term field (MH); search title field (TI); explode (+)

**Appendix Table 2:** *Indicative data items*

| Citation Details | Study characteristics | Population characteristics | Assessment of PD | Assessment of MSDs | Main results | Reviewer notes |
| --- | --- | --- | --- | --- | --- | --- |
| Study ID: | Aims: | Age (SD/range): | PD diagnosis (e.g. any; Cluster; specific): | Diagnosis (e.g. arthritis): | Critical appraisal score: | Data of data extraction: |
| Authors: | Study design: | Sample size: | Probable PD diagnosis (e.g. any; borderline PD): | Diagnostic method (e.g. expert/self-report): | Unadjusted Odd Ratio (OR) and 95% Confidence Intervals (95% CI): | Reviewer who performed the data extraction: |
| Journal: | Sample Representativeness: | Age groups: | Diagnostic criteria (e.g. DSM-IV, DSM-IV/TR, DSM-5, ICD-10): | Diagnostic criteria/definition: | Adjusted OR and (adjusted for): |  |
| Year: | Year/ timeframe for data collection: | Sex: | Assessment tool (e.g. SCID-II; IPDE-screener: |  | % with/without PD in sample: |  |
| Pages: | Method of data analysis: | Socio-economic status indicator: | Administration of tool (expert; trained personnel; self-reported): |  |  |  |
|  |  |  | Psychiatric comorbidity (major depressive episode, major depressive disorder): |  |  |  |
|  |  |  |  |  | % with/without MSD in sample: |  |
|  | Confounders/covariates examined: |  |  |  | Stratified sample/sub-group results (sex; age group; condition): |  |

*Note:* Shaded cells represent key data items to be prioritized for extraction

**Appendix Figure 1:** *Project timeline in 2023*

*
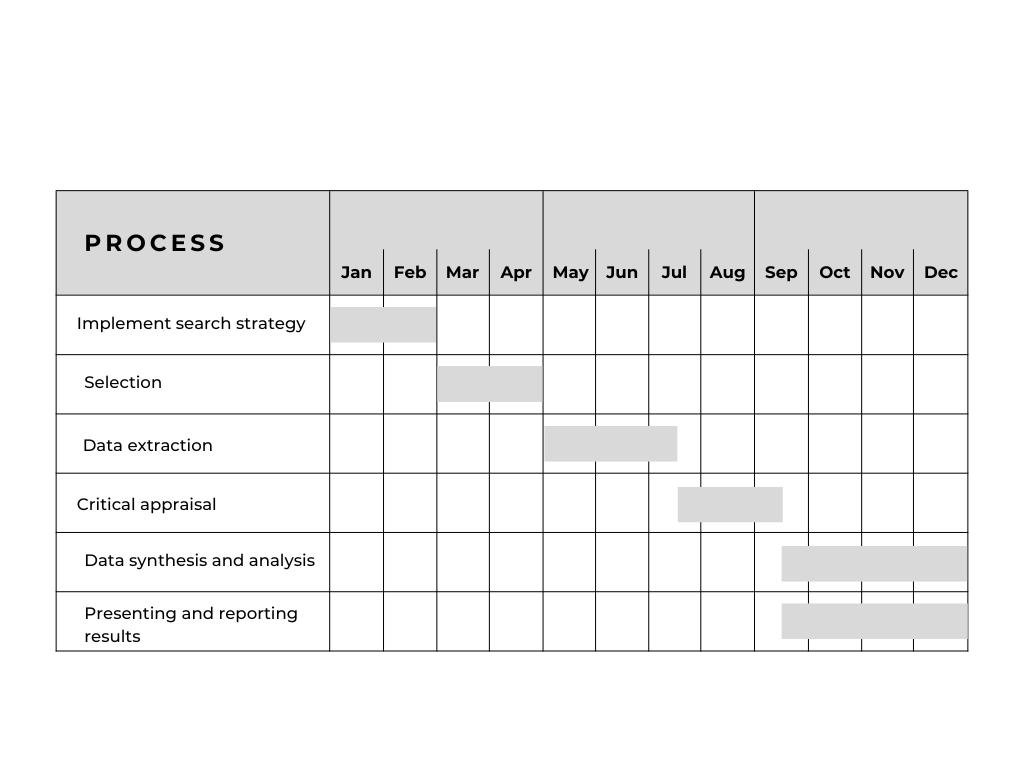
*
